# Endemic treponemal diseases

**DOI:** 10.1093/trstmh/tru128

**Published:** 2014-08-25

**Authors:** Michael Marks, Anthony W Solomon, David C Mabey

**Affiliations:** a Clinical Research Department, Faculty of Infectious and Tropical Diseases, London School of Hygiene & Tropical Medicine, Keppel Street, London, WC1E 7HT, UK; b Hospital for Tropical Diseases, University College London Hospitals NHS Trust, London, WC1E 6JB, UK

**Keywords:** Bejel, Neglected tropical diseases, Pinta, Syphilis, Yaws

## Abstract

The endemic treponemal diseases, consisting of yaws, bejel (endemic syphilis) and pinta, are non-venereal infections closely related to syphilis, and are recognized by WHO as neglected tropical diseases (NTDs). Despite previous worldwide eradication efforts the prevalence of yaws has rebounded in recent years and the disease is now a major public health problem in 14 countries. Adequate data on the epidemiology of bejel and pinta is lacking. Each disease is restricted to a specific ecological niche but all predominantly affect poor, rural communities. As with venereal syphilis, the clinical manifestations of the endemic treponemal diseases are variable and can be broken down in to early stage and late stage disease. Current diagnostic techniques are unable to distinguish the different causative species but newer molecular techniques are now making this possible. Penicillin has long been considered the mainstay of treatment for the endemic treponemal diseases but the recent discovery that azithromycin is effective in the treatment of yaws has renewed interest in these most neglected of the NTDs, and raised hopes that global eradication may finally be possible.

## Introduction

The endemic treponemal diseases, consisting of yaws, bejel (endemic syphilis) and pinta, are non-venereal infections closely related to syphilis, and are recognized by WHO as neglected tropical diseases (NTDs).^1^ Each disease is caused by an organism of the genus *Treponema;* yaws by *T. pallidum* subspecies *pertenue*, bejel by *T. p.* subsp. *endemicum*, and pinta by *T. carateum,* and each occupies a limited geographical and ecological niche. The prevalence of all three diseases is thought to have declined substantially in the last 100 years, although there are limited recent data from much of the world.

All three diseases are characterized by multi-stage infection predominantly involving skin, bones and cartilage. The diseases vary in the incidence and severity of late stage disease. As with syphilis, penicillin has been the mainstay of treatment,^2^ but azithromycin has recently been shown to be effective in the treatment of yaws,^3^ prompting renewed efforts to eradicate it.^4^

## Epidemiology

**Figure 1.**
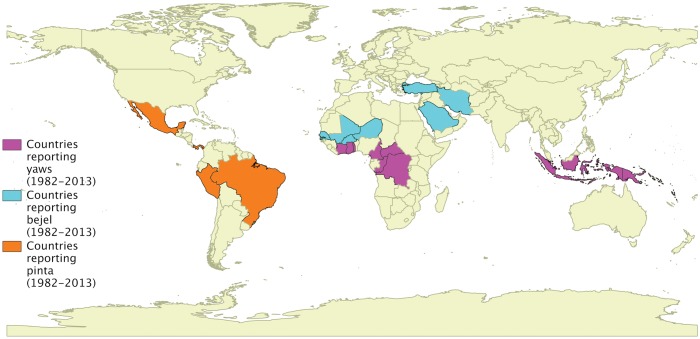
Countries where endemic treponemal diseases have recently been reported. This figure is available in black and white in print and in color at Transactions online.

Based on current reports, yaws is thought to be the most prevalent of the endemic treponemal diseases, but it is likely that there is significant under-reporting of all three diseases (Figure 1).

In the mid-twentieth century, as many as 50 million individuals were thought to be infected by yaws.^5^ In 1949, soon after WHO was established, the World Health Assembly passed a resolution to support the control and elimination of the endemic treponematoses. Between 1952 and 1964 WHO and UNICEF coordinated a global treatment programme for the control of endemic treponemal diseases which was thought to have reduced the global burden by as much as 98%.^5^ The disease subsequently rebounded in a number of countries in the 1970s, and a second World Health Assembly resolution was passed in 1978 leading, in some places, to renewed control efforts.^6^ Despite these efforts the disease was not eliminated and the number of cases reported worldwide has continued to climb in recent years.

Yaws is currently thought to be endemic in 14 countries, mostly in West Africa, South East Asia and the Pacific.^7–12^ Papua New Guinea, the Solomon Islands and Ghana have each reported more than 20 000 cases of yaws in recent years. Yaws was previously reported to be endemic in South America and the Caribbean, but control programmes in the mid-twentieth century are thought to have successfully eliminated yaws from most countries in the region except Guyana.^13,14^ India interrupted transmission in 2004 and declared elimination in 2006^15^ following a sustained programme which began in 1996. Endemic countries report cases of yaws annually to WHO. Many countries report clinical cases without serological confirmation. Integration of confirmatory diagnostic tests in to national reporting structures will be needed to support the current WHO yaws eradication program.

Pinta is restricted to Latin America, in particular Mexico and Colombia,^16,17^ although there are case reports of it occurring in Cuba where it was previously thought to have been eliminated.^18^ Bejel has been reported from both the Sahel region of Africa and the Arabian peninsula, and was previously reported to be endemic in the Bedouin population of Saudi Arabia;^19,20^ recent reports suggest ongoing transmission in isolated, rural populations.^21^ Cases of all three diseases in non-endemic countries have been reported in immigrant populations.^18,22,23^ Except for these case reports there are almost no recent systematic data on the prevalence of either bejel or pinta in many countries where they were previously thought to be of public health significance, which is likely to reflect in part the difficulty in reaching the isolated communities affected by these diseases. The collection of accurate prevalence data is a high priority for control efforts for both of these diseases.

Yaws and pinta are both found in warm and humid environments,^24^ whilst bejel is found in drier climes.^25,26^ All three diseases are thought to be spread via direct skin to skin contact,^2^ whilst indirect contact via shared objects such as utensils has been additionally implicated in the transmission of bejel.^25,26^ Flies have been postulated to mechanically transfer yaws and pinta^2^ but there is no definitive proof that this occurs. Whilst adults are the main reservoir of venereal syphilis, young children and their contacts act as the reservoirs of yaws and bejel, whilst active cases, predominantly in young adults, are thought to be the main reservoir of pinta.^2^ Closely related treponemal infections have been identified in primate populations, but zoonotic transmission to humans has not been established.^27^ Whilst HIV has been associated with a higher rate of atypical clinical lesions in syphilis, there are no published data on the impact of HIV on the endemic treponematoses.

## Organism

Treponemes are gram-negative spirochetes which cannot be cultured in vitro.^2^ The causative organisms of the endemic treponemal diseases cannot be differentiated from *T. p.* subsp. *pallidum* by morphology, physiology or host response.^28^ Only a limited number of treponemal strains have been fully sequenced due to the traditional requirement for primary isolation of organisms in rabbits.^28^ Furthermore there are no currently available strains or samples of *T. carateum* available for sequencing.^29^ Genome data that are available demonstrate that the causative agents of yaws and bejel are closely related to *T. p.* subsp. *pallidum*,^28^ with the genomes of *T. p.* subsp. *pallidum* and *T. p.* subsp. *pertenue* differing by less than 0.2%.^28^ Genome analysis has been used to inform studies on the phylogentic relationship of treponemes but, given the limited number of strains sequenced, their precise relationships remain unclear.^29^

## Pathology and pathogenesis

Insights into the pathogenesis of yaws have been predominantly derived from studies in hamster and rabbit models of disease, which suggest significant overlap in the pathogenesis of all the treponemal diseases.^30^ Infection is thought to be spread by skin to skin contact when bacteria from a lesion enter via a breach in the skin,^2,31^ or via indirect transmission by shared utensils in the case of bejel.

In animal models of yaws, the minimum infective dose has been found to be 10^3^–10^4^ organisms,^32^ and the rate at which lesions appear and subsequently resolve has been shown to be related to the size of the inoculum.^32^ Following infection, treponemes disseminate to lymph nodes where they multiply rapidly.^32^

The host response to infection is mediated by both cellular and humoral immune responses. In animal models, passive transfer of immune serum at the time of inoculation protects against the development of clinical disease.^33,34^ A number of strategies have been suggested by which *T. pallidum* might evade the immune system to establish chronic infection, including depressing the response of lymphocytes to mitogens, antigenic variation, and the organism's extremely low metabolic rate which allows it to avoid immune recognition during latent infection.^35,36^

## Clinical features

As with venereal syphilis, the endemic treponemal infections are all capable of causing chronic disease, which is often classified as either early or late.^2^ There is considerable overlap in the early clinical features of the endemic treponemal diseases, but there are important differences in the appearance and frequency of late stage manifestations. The manifestations of all three may be confused with syphilis.^37^

### Yaws

**Figure 2.**
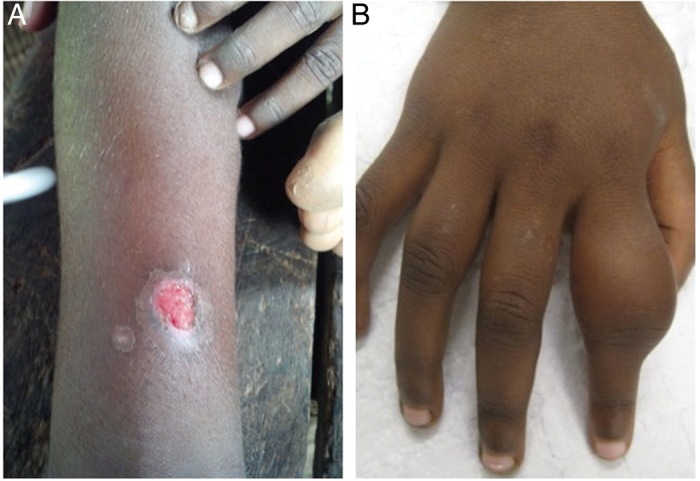
Lesions of yaws. (A) Ulcer of primary yaws. (B) Dactylitis of secondary yaws. Figure 2A courtesy of M. Marks. Figure 2B courtesy of O Mitjà. This figure is available in black and white in print and in color at Transactions online.

Primary yaws is characterized by the development of an erythematous lesion^2^ at the site of inoculation, which may break down^16^ to form an ulcerating plaque over a period of 1–2 weeks (Figure 2). These lesions most commonly occur on the lower limbs or buttocks^24,38^ after an incubation period of 9–90 days.^2,16^ Genital lesions are extremely uncommon. In the absence of treatment, lesions heal spontaneously, with scarring, within 3–6 months.^39^

Untreated individuals may go on to develop secondary yaws, which predominantly affects the skin and bones.^38,40^ Generalized lymphadenopathy and constitutional symptoms are also frequently reported in patients with secondary yaws.^16^ A variety of skin manifestations of secondary yaws have been described, including multiple ulcerative lesions, macular lesions, and hyper-keratotic lesions on the palms and soles (‘crab yaws’).^2,16,38^ Bone disease is most commonly manifested as osteoperiostitis, affecting the fingers (resulting in dactylitis) or long bones (forearm, fibula, tibula) and resulting in bone pain and swelling.^40^ In most patients, multiple bones are affected. Following treatment of primary or secondary yaws, skin lesions usually resolve within 2–4 weeks and bone pain may begin to resolve in as little as 48 hours.^41^

As with venereal syphilis, untreated patients may develop latent infection, with only reactive serology as evidence of their having acquired infection. Reactivation from the latent state can occur up to several years later.

Tertiary yaws was previously reported to occur in about 10% of untreated individuals,^16^ but is now rarely seen. The disease may manifest as gummatous nodules causing tissue necrosis, a destructive osteitis which may cause bowing of the shins (sabre shin) or destruction of the maxilla (gangosa), or a hypertrophic periostitis causing exostosis of the paranasal maxilla (gondou). Although there are anecdotal reports, there is no definitive evidence that neurological or cardiovascular manifestations of yaws occur.^2^

### Bejel

**Figure 3.**
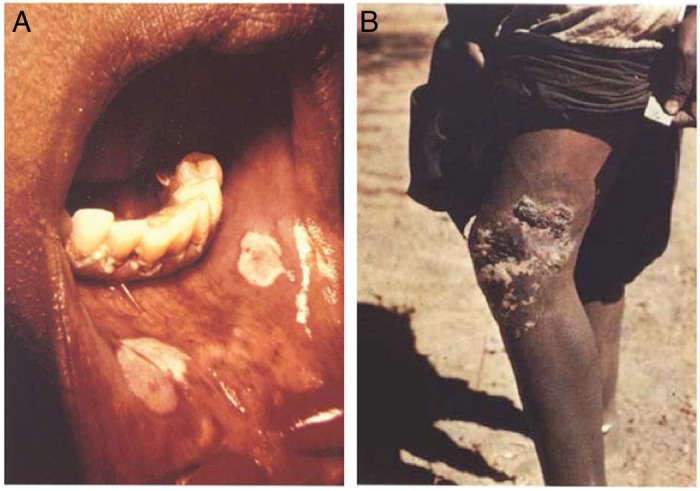
Lesions of bejel. (A) Oral lesion of primary bejel. (B) Chronic skin lesion of secondary bejel. Figure reproduced from Perine et al.^2^ with permission of the publisher. This figure is available in black and white in print and in color at Transactions online.

The initial lesions of primary bejel manifest as small, painless ulcers of the mucus membranes of the oropharynx and nasopharnyx^20,42^ (Figure 3). Due to their small size and location, the primary lesion is often not clinically apparent until secondary lesions develop, typically 3 to 6 months after infection. Secondary disease is characterized by lesions of the mucus membranes, accompanied by regional lymphadenopathy, a diffuse maculo-papular rash and condylomata lata, which may be mistaken for secondary syphilis. As in yaws, secondary bejel may be accompanied by osteitis and periosteitis resulting in painful bony swelling.^42,43^

Untreated bejel progresses to late stage disease which is characterized by gummatous lesions. Gummatous nodules of the skin can progress to become infiltrated, abnormally pigmented lesions,^21,43^ whilst gummata affecting the nasopharnyx can result in a destructive rhinopharyngitis (gangosa), which is also seen in tertiary yaws. In contrast to venereal syphilis, cardiovascular and neurological manifestations do not occur in tertiary bejel.

### Pinta

**Figure 4.**
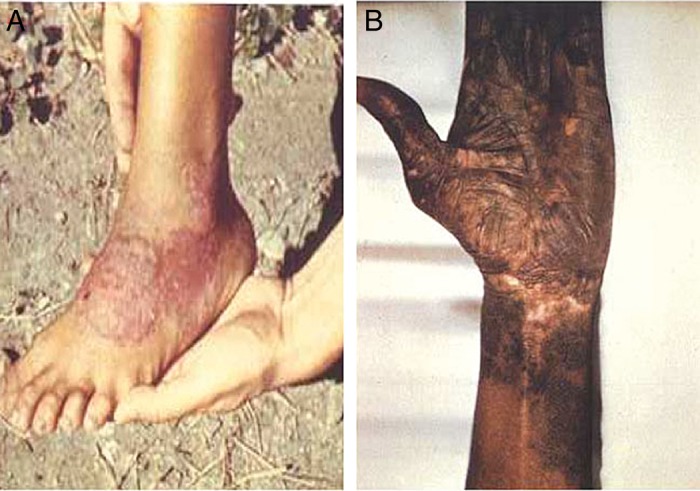
Lesions of pinta. (A) Erythematous plaque of early pinta. (B) Hyperpigmented skin lesion of late pinta. Figure reproduced from Perine et al.^2^ with permission of the publisher. This figure is available in black and white in print and in color at Transactions online.

Pinta is considered the most benign of the endemic treponemal diseases, with all manifestations limited to the skin^2^ (Figure 4). The initial lesions of pinta form as papules or erythematous plaques which may become pigmented and hyperkeratotic, and are often accompanied by regional lymphadenopathy.^44^ Lesions are most commonly found on exposed areas of skin, in particular on the lower limbs and forearms.^2,45^ Constitutional symptoms do not accompany infection with pinta.

Early stage lesions normally resolve spontaneously^42^ but are followed several months later by the appearance of multiple smaller lesions (‘pintids’) which may be associated with alterations in skin pigmentation.^42^ These smaller skin lesions may enlarge and coalesce over time.^2^ Treponemes may continue to be found in pinta lesions for several years, and affected individuals therefore remain infectious.

The later lesions of pinta are characterized by ongoing pigmentary changes which may manifest as both hyperpigmentation and hypopigmentation. Hyperpigmentation can vary from red, blueish violet to brown.^42^ Both hyper and hypopigmentation may occur within the same lesion and at different rates. Pigmentary changes may be accompanied by both skin atrophy and hyperkeratosis.^2,42^ Unlike the other treponematoses, in pinta, neither destructive skin and bone lesions nor cardiovascular and neurological manifestations occur.

## Attenuated disease

There are reports that the clinical manifestations of the endemic treponemal diseases are less florid than previously described^10,44^ and that, in particular, tertiary manifestations are now infrequently seen. There is no accepted definition of attenuated disease, nor a clear explanation for why the clinical phenotype of the disease may have altered. Improvements in hygiene, use of treponemocidal antibiotics for other conditions, and mutations in *T. p.* subsp. *pertenue* have all been proposed as contributing to the change in phenotype.^10^

## Differential diagnosis

The differential diagnosis of the endemic treponematoses is broad and varies between the different diseases and disease stages. Syphilis is a key differential diagnosis for all three endemic treponemal infections. The early lesions of yaws may also be confused with bacterial skin infections including tropical ulcer, and parasitic infections including leishmaniasis. Early bejel may be mistaken for oral herpes simplex, aphthous ulceration and syphilis. The later stages of either disease may be mistaken for syphilis, fungal infection, mycobacterial infection, psoriasis and eczema. Early lesions of pinta may be mistaken for eczema, psoriasis, tinea, pellagra, syphilis and leprosy, whilst dyschromic late stage lesions may be confused with vitiligo, leprosy, melisma and fungal infection.^46,47^

## Diagnosis

Pathogenic treponemes cannot be cultured in vitro, which has limited the utility of direct diagnostic methods. As with venereal syphilis, dark field microscopy can be used to identify viable spirochetes from lesion swabs,^2^ although this technique is not routinely available in the settings in which these diseases are prevalent.

As with venereal syphilis, serology remains the cornerstone of diagnosis. The *Treponema pallidum* particle agglutination (TPPA) and haemagglutination (TPHA) assays are used to detect *Treponema*-specific antibodies. Once positive, these tests usually remain positive for life. The venereal disease research laboratory (VDRL) and rapid plasma reagin (RPR) tests are non-specific tests detecting antibodies to cardiolipin.^2^ Although VDRL and RPR tests have low specificity, they more accurately reflect disease activity than TPPA or TPHA, with titres falling rapidly following successful treatment.

Whilst most countries have central laboratory facilities to perform serological testing, access to these facilities may be difficult for the isolated, rural communities most commonly affected by endemic treponemal diseases. Current studies are assessing whether sensitive and specific point of care tests developed for syphilis^48^ may be of value in the diagnosis and management of endemic treponemal diseases. Initial results of these studies suggest that these tests may be of value in the diagnosis of yaws.^49^

Current serological techniques cannot distinguish infection with syphilis from infection with any of the non-venereal trepanomatoses.^50^ Given the considerable epidemiological overlap between venereal syphilis and the endemic treponemal diseases, interpretation of serology currently depends on an understanding of the epidemiology and clinical manifestations of each disease.

Whilst PCR-based assays have become routinely available for the diagnosis of syphilis^51^ in high-income countries, there is limited access to such technology in low-income settings. Current assays cannot distinguish subspecies of pathogenic treponemes, but combined PCR and DNA sequencing has been used to confirm the diagnosis in imported cases of both yaws and bejel.^22,23^ These techniques are currently only available at a small number of reference laboratories.

## Treatment

Intramuscular benzathine penicillin was the mainstay of treatment for both venereal syphilis and the endemic treponemal diseases for over 50 years.^2^ For endemic treponemal diseases a dose of 1.2 million units is recommended for patients aged 10 or over, whilst a dose of 0.6 million units is recommended for patients aged under 10 and for contacts. These doses are considerably lower than those recommended for venereal syphilis, and if there is any doubt as to the diagnosis in patients with clinical or serological evidence of a treponemal infection, clinicians in both endemic and non-endemic settings are recommended to use a treatment regimen appropriate for syphilis.

There have been a number of recent reports suggesting penicillin failure in the treatment of yaws in Papua New Guinea,^52^ which the authors attributed to reduced sensitivity of *T. p.* subsp. *pertenue* to penicillin. However the inability to distinguish treatment failure from re-infection makes the implications of these reports unclear.

A recent open label randomized control trial in Papua New Guinea demonstrated that a single oral dose of azithromycin (30 mg/kg, maximum 2G) was non-inferior to penicillin in the treatment of patients with primary or secondary yaws.^3^ Azithromycin has also previously been shown to be effective in the treatment of syphilis, but its use has been hampered by the development of resistance associated with point mutations in the 23 s ribosomal gene.^53^ Azithromycin has not been formally studied in bejel or pinta but is likely to be efficacious in the treatment of these infections.

No other agents have been shown in randomized clinical trials to be effective in the treatment of endemic treponemal diseases, but there are reports of treatment success with treatment regimens based on oral penicillin and tetracycline derivatives,^14,54^ whilst erythromycin has been recommended based on its efficacy in the treatment of syphilis.

## Control programs and elimination

Previous WHO programs in the 1950s–60s successfully reduced the burden of yaws worldwide^5^ but did not successfully eradicate the disease, due in part to a failure to adequately identify and treat contacts and latent cases.^55^ More recently, national programmes have been successful at eliminating the disease from Ecuador^56^ and India.^15^ The success of azithromycin in treating yaws has prompted WHO to renew efforts to eradicate the disease by 2020 using a strategy of community mass treatment.^4^ Yaws is endemic in a number of countries where trachoma is also found. As both diseases are controlled via mass distribution of azithromycin there is a possibility of integrating these control efforts, although further study will be needed given the lower dose of azithromycin used in trachoma control programs.

Whilst there is considerable optimism, there are several areas for concern including the need for more accurate data on the prevalence of yaws in countries where the disease was previously reported to be endemic, and the possibility that resistance to azithromycin will emerge, as it has in syphilis.^53^ The WHO plan will require significant support from NGOs, academic institutions and governments to ensure these issues are addressed and that adequate monitoring is undertaken to ensure that the eradication strategy is successful.

At present WHO has not developed a specific strategy for the control or eradication of either bejel or pinta. Both of these diseases have previously been eliminated in some countries, raising the prospect that they could also be targeted for eradication. Such an undertaking would require significant research to update and improve our understanding of the current epidemiology of these diseases, and to assess treatment strategies.

## Conclusions

The endemic treponemal diseases remain important problems in the poor, rural communities in which they are found, and place a considerable burden on local health care systems. The diseases share a number of common features including a multi-stage disease process, serological findings and response to treatment, but differ in the frequency and severity of their late manifestations. Whilst penicillin has long been considered the mainstay of treatment, a recent study demonstrating the efficacy of azithromycin in the treatment of yaws has raised hopes that global eradication of these diseases may finally be possible.
